# Mapping the frontiers: a bibliometric perspective on t cell-based immunotherapy in pancreatic cancer since the twenty-first century

**DOI:** 10.1007/s00432-025-06356-x

**Published:** 2025-11-04

**Authors:** Ziniu Tang, Chen Wang, Zhenyu Ma, Peng Shang, Qipeng Yuan, Jinbo Yue

**Affiliations:** 1 Department of Radiation Oncology, Shandong Cancer Hospital and Institute, Shandong First Medical University, Shandong Academy of Medical Sciences, Jinan, China; 2 Cheeloo College of Medicine, Shandong University Cancer Center, Shandong University, Jinan, Shandong China

**Keywords:** T-cell immunotherapy, Pancreatic carcinoma, Bibliometric analysis, Tumor microenvironment

## Abstract

**Purpose:**

T-cell immunotherapy is reshaping cancer care and offers a targeted strategy for pancreatic carcinoma (PC), yet a comprehensive map of its research trajectory is lacking. We aimed to chart the evolution of the field, identify leading contributors, and clarify the thematic shifts that are shaping clinical translation.

**Methods:**

We systematically analyzed articles and reviews indexed in the Science Citation Index Expanded of the Web of Science Core Collection from January 2000 to December 2024. Bibliographic metadata were aggregated for descriptive trend analyses and science-mapping of co-authorship, co-citation, and keyword co-occurrence networks. Temporal trend profiling was used to highlight emerging topics.

**Results:**

Global output on T-cell immunotherapy for PC has expanded markedly over the past two decades but remains unevenly distributed across regions. The United States leads in academic influence and translational impact, with China closely following in publication volume and contributions from high-impact institutions. Research has converged at the interface of immunotherapy, tumor-microenvironment modulation, and cellular engineering. Dominant themes include engineered T-cell approaches, immune-checkpoint modulation, and strategies leveraging tumor-infiltrating lymphocytes. Emerging fronts encompass AI-enabled target/drug discovery, biomarker-guided patient stratification, and individualized treatment designs. Persisting barriers include limited efficacy in the desmoplastic and immunosuppressive PC microenvironment, primary and acquired immune resistance, safety concerns, and regulatory and trial-design complexities.

**Conclusions:**

T-cell immunotherapy for PC is a rapidly advancing, interdisciplinary domain led by the United States with rising contributions from China. Accelerating clinical translation will require: integrated T-cell engineering with microenvironment remodeling; rational combinations with checkpoint and stroma-targeted agents; robust predictive biomarkers with standardized endpoints; safety-engineering and risk-mitigation frameworks; and coordinated, multicenter collaboration. This bibliometric synthesis delineates the field’s structure and priorities to improve outcomes for patients with PC.

## Introduction

Pancreatic cancer (PC) is one of the most aggressive malignancies of the digestive system and remains a major global health challenge. It is currently the seventh leading cause of cancer-related deaths worldwide, accounting for 4.7% of total cancer mortality, and is projected to surpass breast cancer as the third leading cause of cancer deaths by 2025 (Sung et al. 2021). The insidious onset of PC, coupled with the lack of early symptoms, makes early detection difficult. As a result, more than 80% of cases are diagnosed at an advanced stage, where surgical resection-the only potentially curative option-is often no longer feasible due to extensive local invasion and distant metastasis (Xie et al. 2018; Wu et al. 2022; Kindler 2018). Consequently, the prognosis remains poor, with limited treatment options and low long-term survival rates. Given these challenges, the pathogenesis and therapeutic strategies for PC have remained critical research priorities.

In recent years, immunotherapies that primarily target tumor cells directly have emerged as highly promising strategies for treating PC, offering an alternative to conventional systemic therapies such as chemotherapy and radiotherapy. Immunotherapy harnesses the body’s immune system to recognize and eliminate cancer cells more effectively. Central to this approach are immune cells, which serve as the critical foundation for cancer immunotherapy (Sharma and Allison 2020; Restifo et al. 2012). Specifically, T cells play a key role in antitumor immunity. Their reactivity is driven by their unique T cell receptors (TCRs), which are capable of recognizing specific antigens presented on tumor cells in the context of human leukocyte antigen (HLA; Major Histocompatibility Complex, MHC) molecules (Alcover et al. 2018). Under normal physiological conditions, such as during an infection, antigen-triggered T cell activation through TCRs initiates a strong immune response. This response leads to the generation of effector lymphocytes that can travel throughout tissues to eliminate target antigens, as well as the formation of memory T cells, which provide long-lasting protection in case of future antigen exposure (Kaech and Ahmed 2001). However, in the native tumor environment, T cells often fail to effectively eliminate tumor cells, despite their ability to infiltrate the tumor microenvironment (TME) and recognize tumor-associated epitopes (Boon et al. 2006). This failure is partly due to the immunosuppressive properties of the TME, which hampers T cell function. While some of this lack of response can be attributed to limited antigenicity on the tumor’s part (Galon and Bruni 2019; Gerard et al. 2021; Pai et al. 2020), research using mouse models of chronic antigen stimulation (Im et al. 2016; Siddiqui et al. 2019; Utzschneider et al. 2016) and studies of tumor-infiltrating lymphocytes (TILs) in immunologically "hot" human tumors (Sade-Feldman et al. 2018; Li et al. 2019; Leun et al. 2020) have shown that T cells can acquire a dysfunctional state during cancer progression. This dysfunction impairs their ability to mount effective antitumor responses, enabling tumors to evade immune surveillance (Thommen and Schumacher 2018; Philip and Schietinger 2022).

In PC specifically-widely recognized as an immunologically “cold” malignancy-the TME is characterized by lower tumor mutational burden (TMB) and neoantigenicity than “hot” tumors such as NSCLC; reduced and often spatially excluded CD8⁺ T cell infiltration; and a dense desmoplastic, myeloid-dominant stroma that impairs antigen presentation and reinforces immunosuppression (Goulart et al. 2021). These features help explain the modest activity of single-agent immune checkpoint blockade in unselected PC and motivate combination strategies that increase antigenicity, enhance T cell trafficking/infiltration, and remodel the TME (Wu et al. 2024). Despite these challenges, advancing our understanding of the properties and dynamics of antitumor T cells is crucial to improving the deployment and manipulation of T cell immunity for cancer treatment. Further investigation into overcoming the obstacles that prevent T cells from eradicating tumor cells will be key to optimizing T cell-based immunotherapies for PC.

This study presents a comprehensive structural bibliometric analysis to map the evolving research landscape of T cell-based immunotherapy for PC since the turn of the twenty-first century. By systematically examining key dimensions, including publication distribution trends, international collaboration networks, institutional contributions, thematic developments, interdisciplinary integration, and dissemination patterns across core journals, this analysis offers a multidimensional reconstruction of the field’s progression. Unlike prior bibliometric studies that have broadly addressed PC or immunotherapy in general, this work is uniquely focused on T cell-based immunotherapy within the context of PC. This targeted scope allows us to capture field-specific dynamics, highlight research hotspots, and identify translational opportunities that might otherwise be obscured in broader analyses. The resulting framework not only aids researchers from diverse disciplines in navigating the expansive scope of this rapidly advancing area but also serves as a valuable reference for newcomers, providing insights into emerging trends and identifying promising avenues for future exploration.

## Results

### Scientific output

**Fig. 1:**
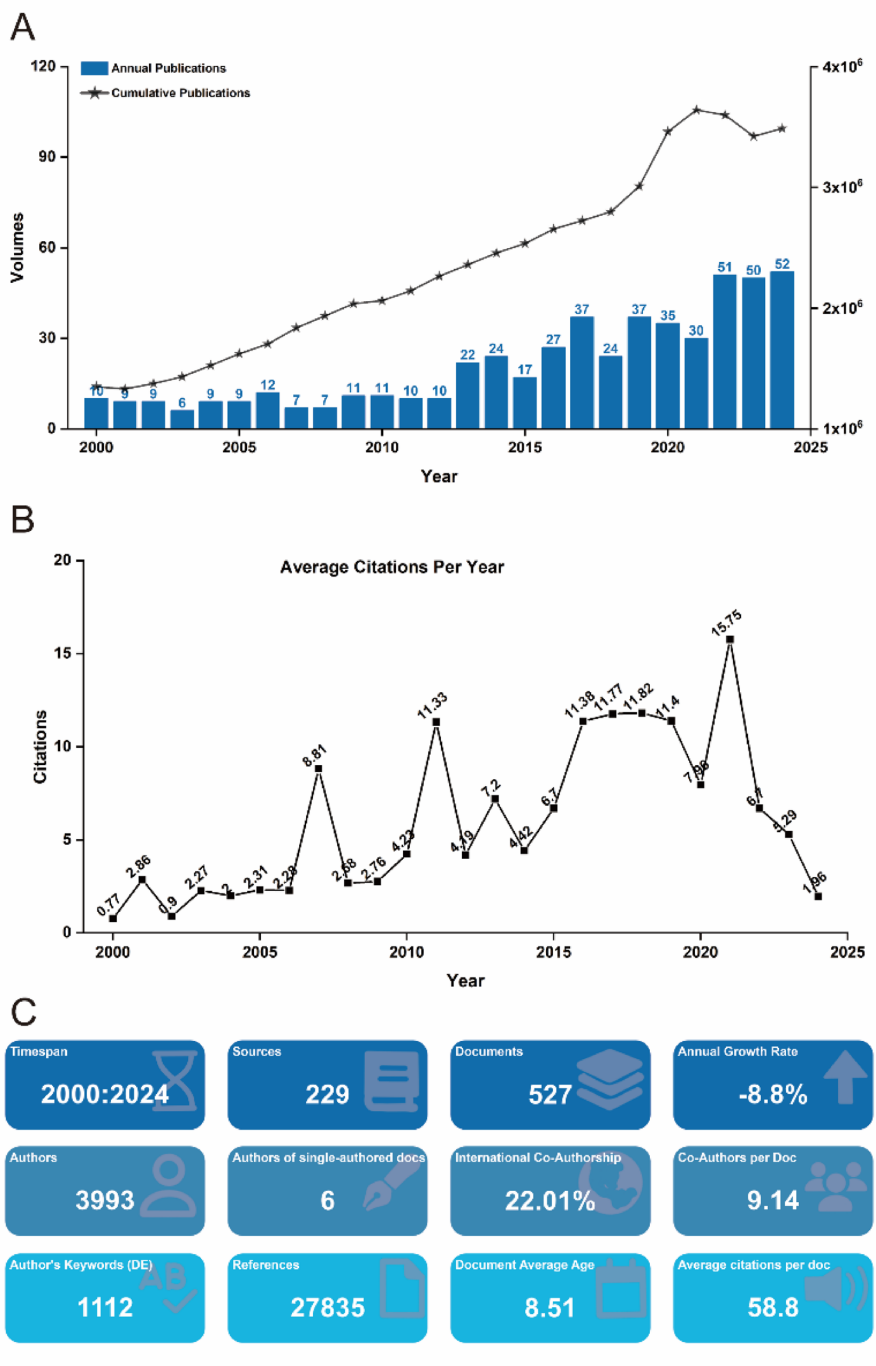
**A** Annual and cumulative output of global T cell-based immunotherapy in PC research publications from 2000 to 2024. **B** Average citations per publication per year from 2000 to 2024. **C** Additional statistics on T cell-based immunotherapy in PC publications from R bibliometrix

Between January 1, 2000, and December 31, 2024, a total of 527 scholarly publications on T cell-based immunotherapy for PC were retrieved from the Web of Science Core Collection (WOSCC), using the search terms “PC” AND “T cell immunotherapy” (Fig. 1A). WOSCC was chosen as the primary data source due to its comprehensive coverage across oncology, immunology, and related fields, as well as its compatibility with bibliometric tools and robust citation data. Following 2017, annual publications consistently exceeded 30, with the last three years seeing more than 50 publications each year, reflecting a notable surge in research activity. The highest annual growth rate was recorded at 41.1%. Citation analysis indicated an average of 5.99 citations per publication per year, with the peak citation impact occurring in 2021, reaching 15.75 citations per publication (Fig. 1B). These trends highlight the growing global attention to T cell-based immunotherapy for PC and underscore the importance of systematically mapping its research landscape.

### Countries/regions

**Fig. 2:**
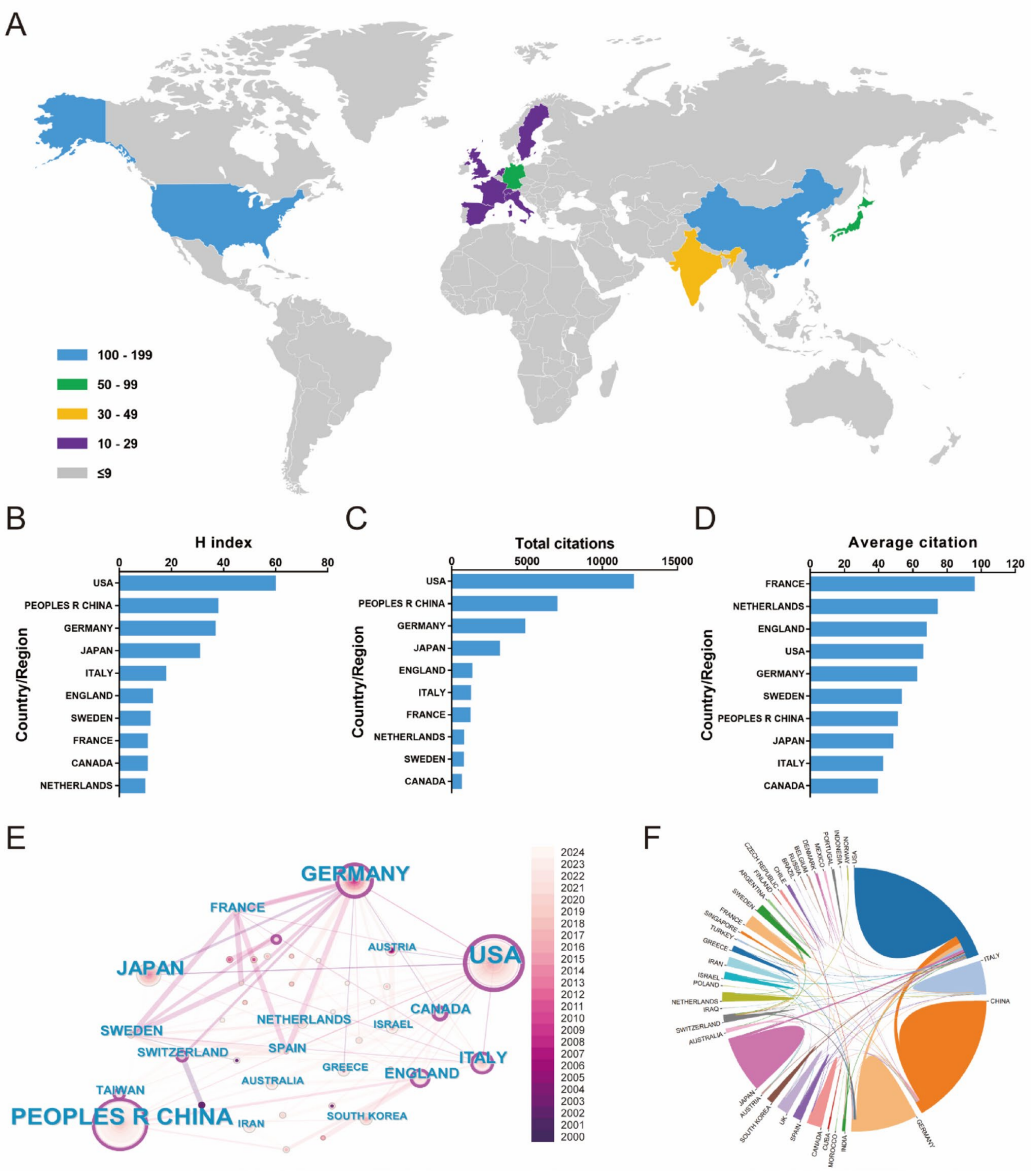
**A** The distribution of T cell-based immunotherapy in PC research from 2000 to 2024. **B**–**D** H index, total citations, and average citation. **E** International collaboration network of 41 countries/regions. Node size represents the number of publications, with larger nodes indicating higher output. The thickness of connecting lines reflects the strength of collaboration (co-authorship frequency) between countries/regions, while color coding corresponds to the average year of publication (as shown in the color scale on the left). Countries with strong centrality values are highlighted with thicker outer rings. **F** Country/region collaboration chord diagram. Each arc represents a country/region, and the width of the arc corresponds to its publication volume. The connecting ribbons illustrate bilateral or multilateral research collaborations, with ribbon width proportional to the intensity of cooperative links

A total of 41 countries/regions had contributed to global scientific productivity in the field of T cell-based immunotherapy for PC, with geographic contributions visualized through a publication density map (Fig. 2A). Table 1 listed the top 10 countries/regions with the highest publication volumes. The USA led with 183 publications, followed by China (137) and Germany (78). The bibliometric analysis highlighted a significant global expansion in this research area, with the USA and China dominating in terms of publication volume, H-index, and total citations (Fig. 2B, C). Countries such as France and Netherlands stood out for their high average citations per publication, reflecting the strong impact of their research (Fig. 2D). International collaboration was extensive, with key research networks connecting USA, Germany, Japan, and China (Fig. 2E, F). These patterns underscored a thriving, interconnected research community that continues to advance the field of immunotherapy for PC.

**Table 1:** Top 10 productive countries/regions in T cell-based immunotherapy in PC, ranked by the number of publications

Rank	Country/Region	Article counts	Percentage of total (%, N/885)
1	USA	183	34.725
2	China	137	25.996
3	Germany	78	14.801
4	Japan	66	12.524
5	Italy	30	5.693
6	England	20	3.795
7	Canada	17	3.226
8	Sweden	15	2.846
9	France	13	2.467
10	Netherlands	11	2.087

### Institutions

**Fig. 3:**
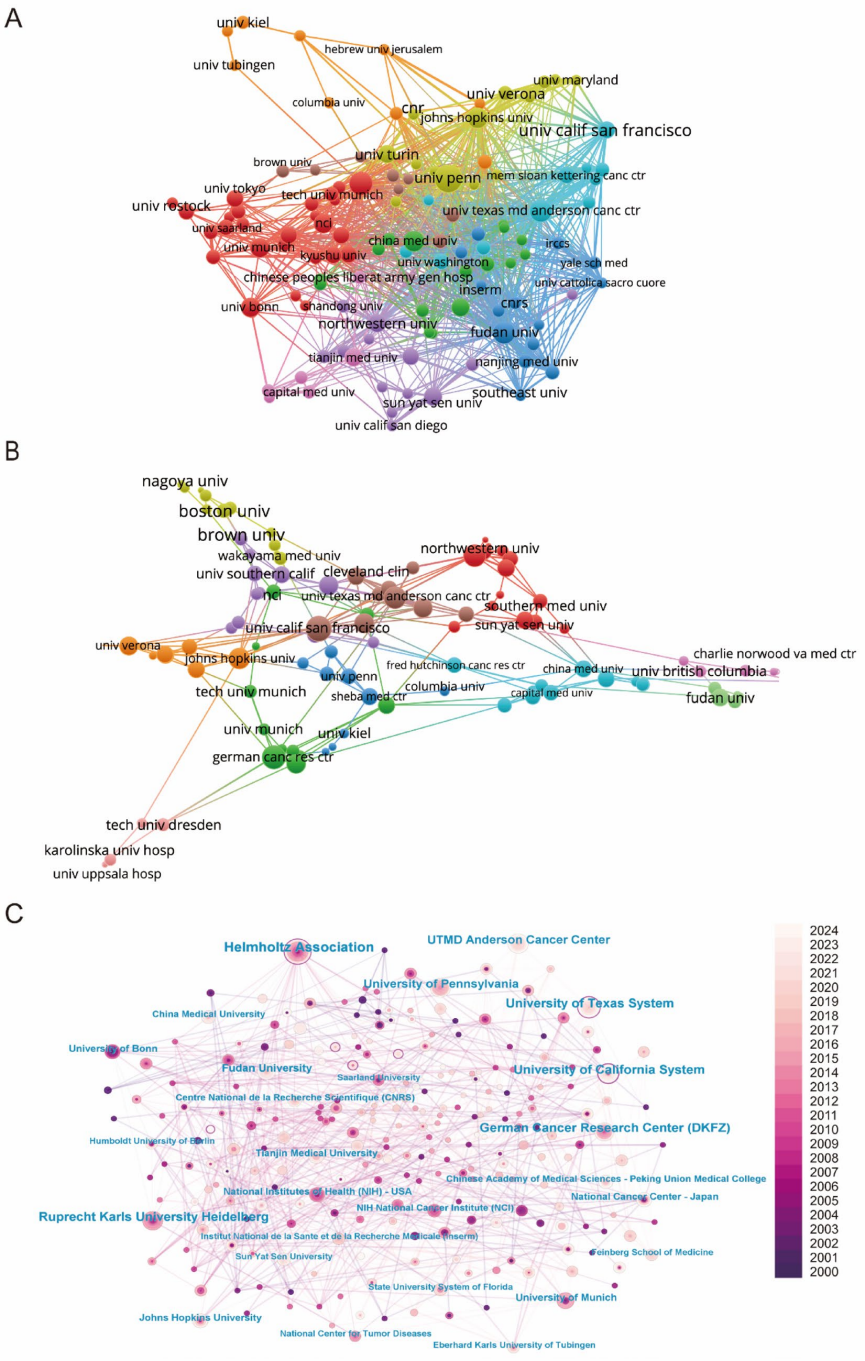
**A** Analysis of country/regional collaboration using Vosviewer. **B** Analysis of institutional collaboration using Vosviewer. **C** Institution network visualization produced using CiteSpace

The global research landscape comprised over 980 participating institutions, as visualized through comprehensive institutional mapping (Fig. 3). The publication leaders were detailed in Table 2, with the Helmholtz Association ranked first, contributing 26 publications, followed by the German Cancer Research Center with 20 publications, and Ruprecht-Karls University Heidelberg with 19 publications. Bibliometric analysis utilizing VOSviewer software revealed distinct dimensions of scientific influence (Figs. 3A, B). Collaborative network mapping (Fig. 3A) identified the USA, Germany, and China as the central knowledge hubs, playing key roles in driving research forward. Citation network analysis (Fig. 3C) highlighted that Helmholtz Association, University of Pennsylvania, and University of Texas System were the central institutions within this network, underlining their dominant positions in advancing research. Temporal network visualization tracked the progressive expansion of research contributions, with institutions such as Helmholtz Association, German Cancer Research Center, and University of California System emerging as central nodes. Notably, recent years had seen a significant increase in research contributions, particularly from China and the USA, underscoring the growing global importance of these institutions as the field continues to evolve.

**Table 2:** Top 10 institutions published literature related to the field of T cell-based immunotherapy in PC from 2000 to 2024, ranked by the number of publications

Rank	Institution	Article counts	Percentage of total (%, N/527)	Country
1	Helmholtz association	26	4.934	Germany
2	German cancer research center	20	3.795	Germany
3	Ruprecht-karls-university heidelberg	19	3.605	Germany
4	University of california system	19	3.605	USA
5	University of texas system	19	3.605	USA
6	University of pennsylvania	18	3.416	USA
7	UT MD anderson cancer center	15	2.846	USA
8	University of bonn	14	2.657	Germany
9	National institutes of health (NIH)	13	2.467	USA
10	University of munich	13	2.467	Germany

**Fig. 4:**
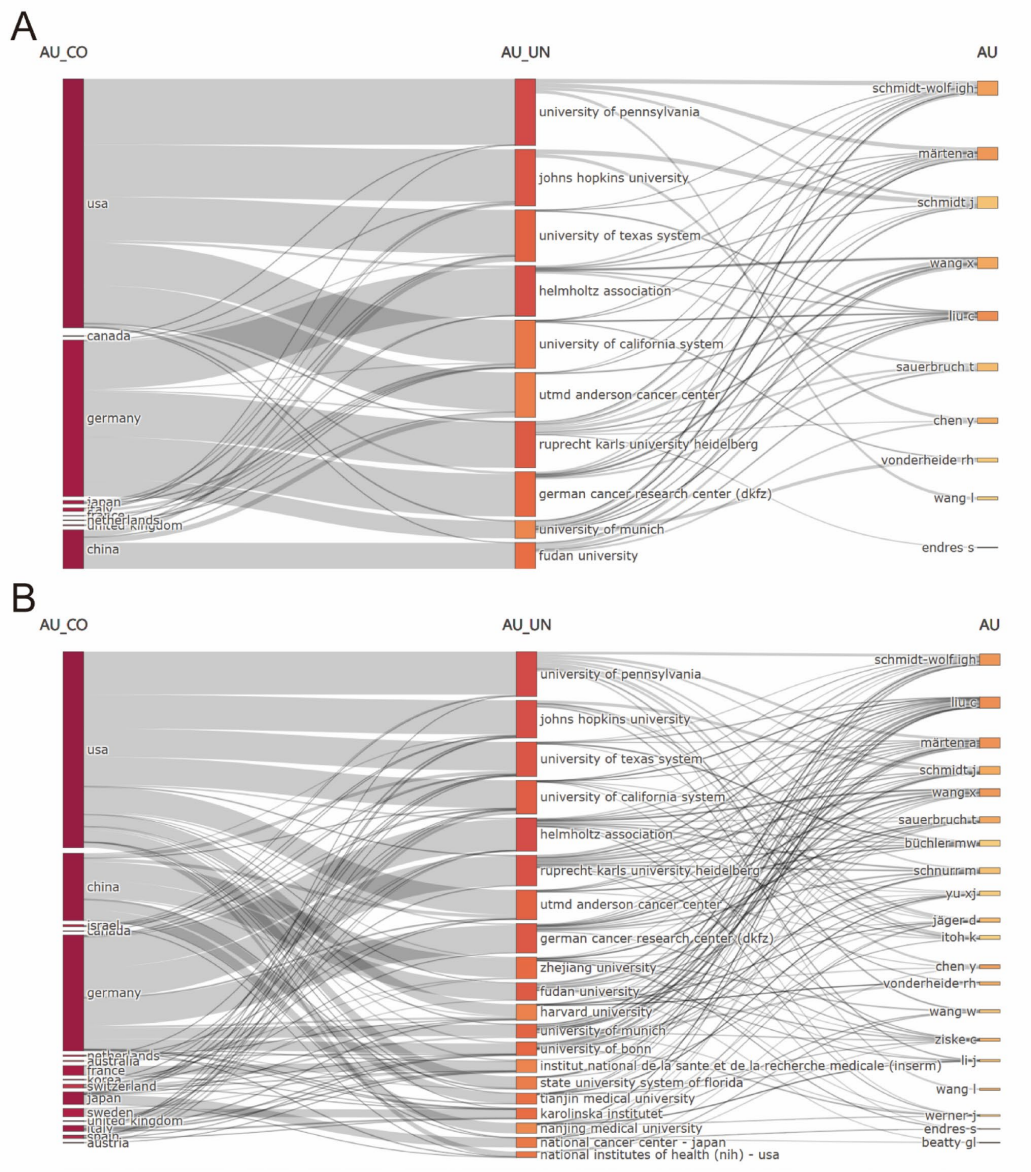
**A** Interconnections of the top 10 high-productivity countries/regions, institutions, and authors. **B** Interconnections of the top 20 high-productivity countries/regions, institutions. and authors. In both panels, the width of each rectangle reflects the number of publications contributed by that entity, while the thickness of the connecting bands indicates the strength of collaborative links between countries, institutions, and authors. The color shading of rectangles (from dark red to orange) corresponds to relative productivity, with darker shades indicating higher publication counts

The interconnection diagrams in Fig. 4 offered valuable insights into the global research ecosystem of T cell-based immunotherapy for PC. Figure 4A revealed the dominant countries, institutions, and authors, with USA, Germany, and China leading in terms of both research output and collaborations. Prominent institutions such as University of Pennsylvania, Johns Hopkins University, and Helmholtz Association occupied central positions, supported by influential authors like Schmidt-Wolf and Matern. Figure 4B expanded the analysis to the top 20 high-productivity countries, regions, and authors, highlighting a broader, interconnected global network. In this fig., AU_CO denotes author collaboration networks, AU_UN reflects author-institution linkages, and AU represents individual author productivity and influence. This extended network illustrated the growing international collaboration, with contributions from Japan, Sweden, Canada, and institutions such as Zhejiang University and Harvard University. The network analysis underscored the increasing scope and collaborative nature of research in T cell-based immunotherapy in PC, emphasizing the role of interconnected academic ecosystems in driving progress in PC treatment.

### Journals

**Fig. 5:**
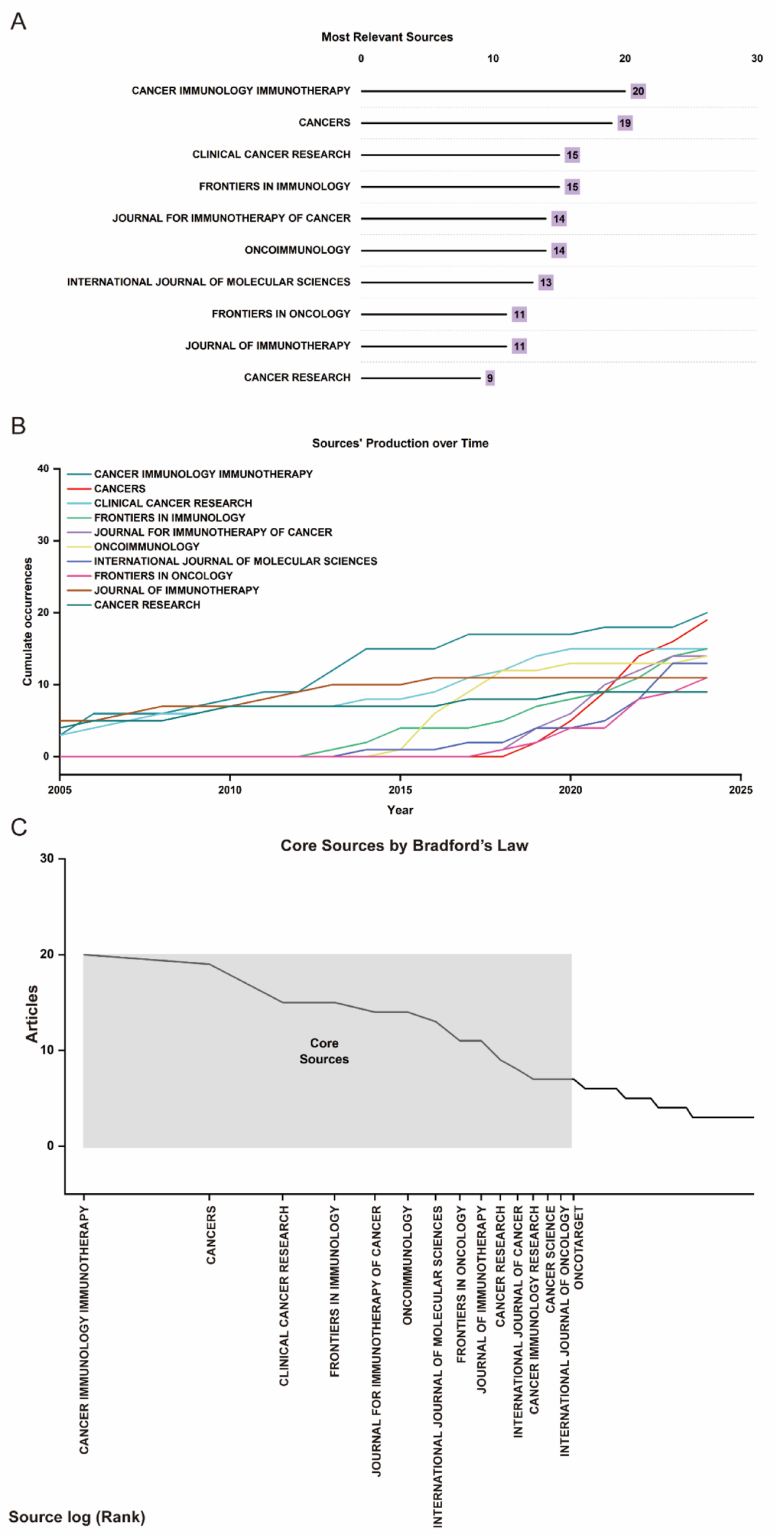
**A** Top 10 journals in T cell-based immunotherapy in PC. **B** Journal output trends within the top 10 from 2000 to 2024. **C** Delineation of core and noncore journals according to Bradford’s law

The bibliometric analysis of T cell-based immunotherapy for PC highlights key trends in publication sources and their evolution over time. Figure 5A identified the top 10 journals in the field, with Cancer Immunology, Immunotherapy leading in terms of publication volume, followed by Cancers and Clinical Cancer Research. Figure 5B illustrated a marked increase in journal output, especially post-2015, indicating a surge in research activity and interest in this area. Figure 5C, based on Bradford’s Law, distinguished core journals that make the most significant contributions to the literature, with the core sources representing the majority of relevant publications in the field. Table 3 provided a detailed ranking of the 19 core journals, with Cancer Immunology, Immunotherapy and Cancers at the top. Table 4 classified 227 journals into three zones, revealing that a small number of core journals dominate the field, while a larger number of secondary journals also make substantial contributions. These findings underscored the centrality of a few high-impact journals while highlighting the diverse range of journals that collectively shape the research landscape of T cell-based immunotherapy for PC.

**Table 3:** Nineteen core source journals in zone 1 according to Bradford’s law

Rank	Journal	Article counts
1	Cancer immunology immunotherapy	20
2	Cancers	19
3	Clinical cancer research	15
4	Frontiers in immunology	15
5	Journal for immunotherapy of cancer	14
6	Oncoimmunology	14
7	International journal of molecular sciences	13
8	Frontiers in oncology	11
9	Journal of immunotherapy	11
10	Cancer research	9
11	International journal of cancer	8
12	Cancer immunology research	7
13	Cancer science	7
14	International journal of oncology	7
15	Oncotarget	7

**Table 4:** According to Bradford’s law, the 227 journals were classified into zones 1–3

Zone	No. of journals	No. of publications	Percentage
1	15	177	33.58%
2	56	177	33.58%
3	156	173	32.82%
Total	227	527	100%

### Subject categories

**Fig. 6:**
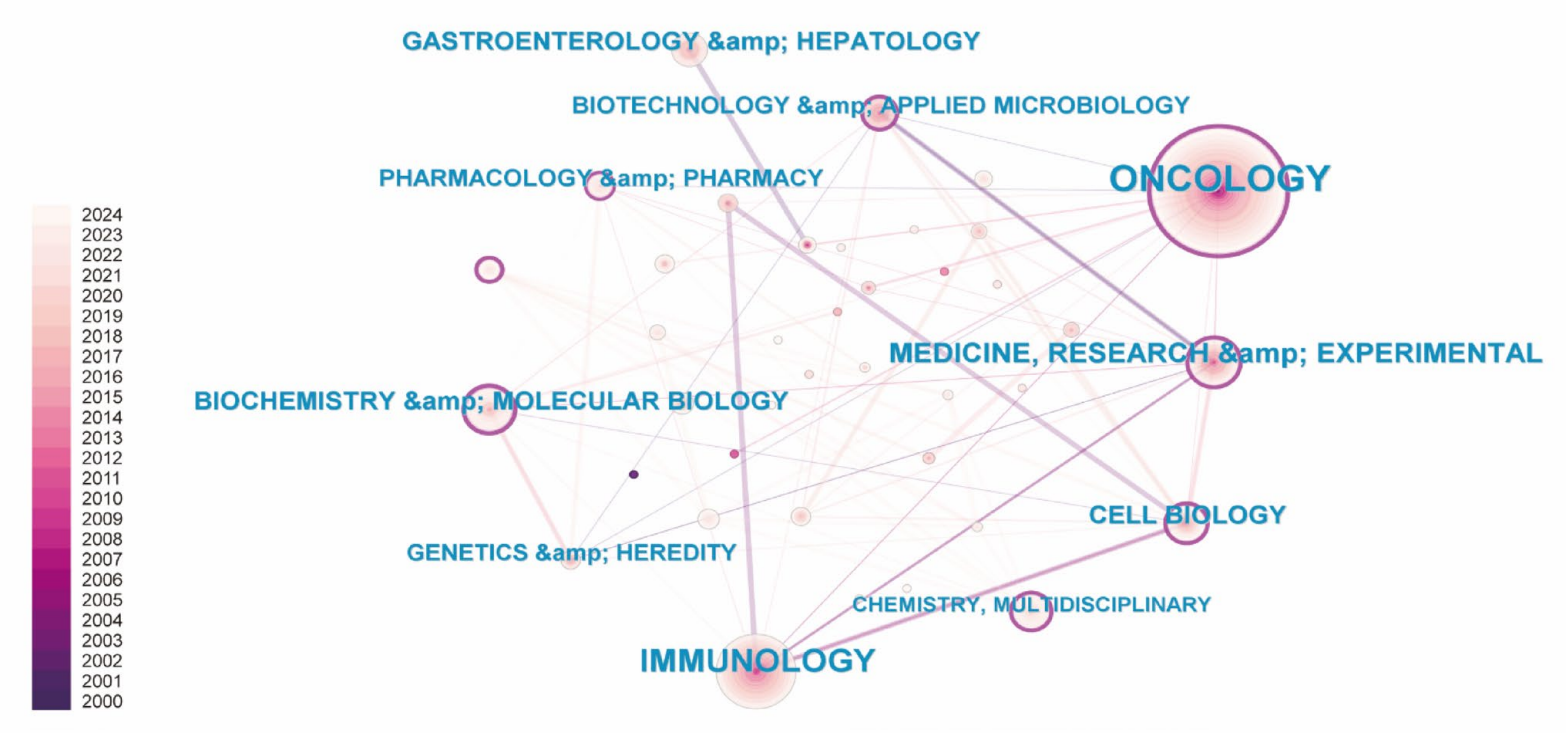
The research category visualization produced using CiteSpace

Figure 6A provided a detailed visualization of the multidisciplinary nature of this research, with oncology at the core and neighboring fields such as immunology, biochemistry, and gastroenterology contributing significantly to the growth of immunotherapy research. The color-coded network showed the rising influence of oncology and immunology, particularly in recent years, reflecting the increasing focus on these disciplines. Table 5 listed the top 10 subject categories by publication volume, with oncology leading at 280 publications, followed by immunology at 122 publications. This data underscored the interconnected and expanding nature of research in T cell-based immunotherapy in PC, with key contributions from biological, clinical, and molecular disciplines.

**Table 5:** Top 10 productive subject categories in T cell-based immunotherapy in PC, ranked by the number of publications

Rank	Subject category	Count	Year
1	Oncology	280	2000
2	Immunology	122	2000
3	Medicine, research & experimental	63	2000
4	Gastroenterology & hepatology	41	2000
5	Biochemistry & molecular biology	40	2002
6	Cell biology	39	2002
7	Biotechnology & applied microbiology	30	2000
8	Pharmacology & pharmacy	29	2000
9	Genetics & heredity	27	2000
10	Chemistry, Multidisciplinary	20	2014

## References and funds

**Fig. 7:**
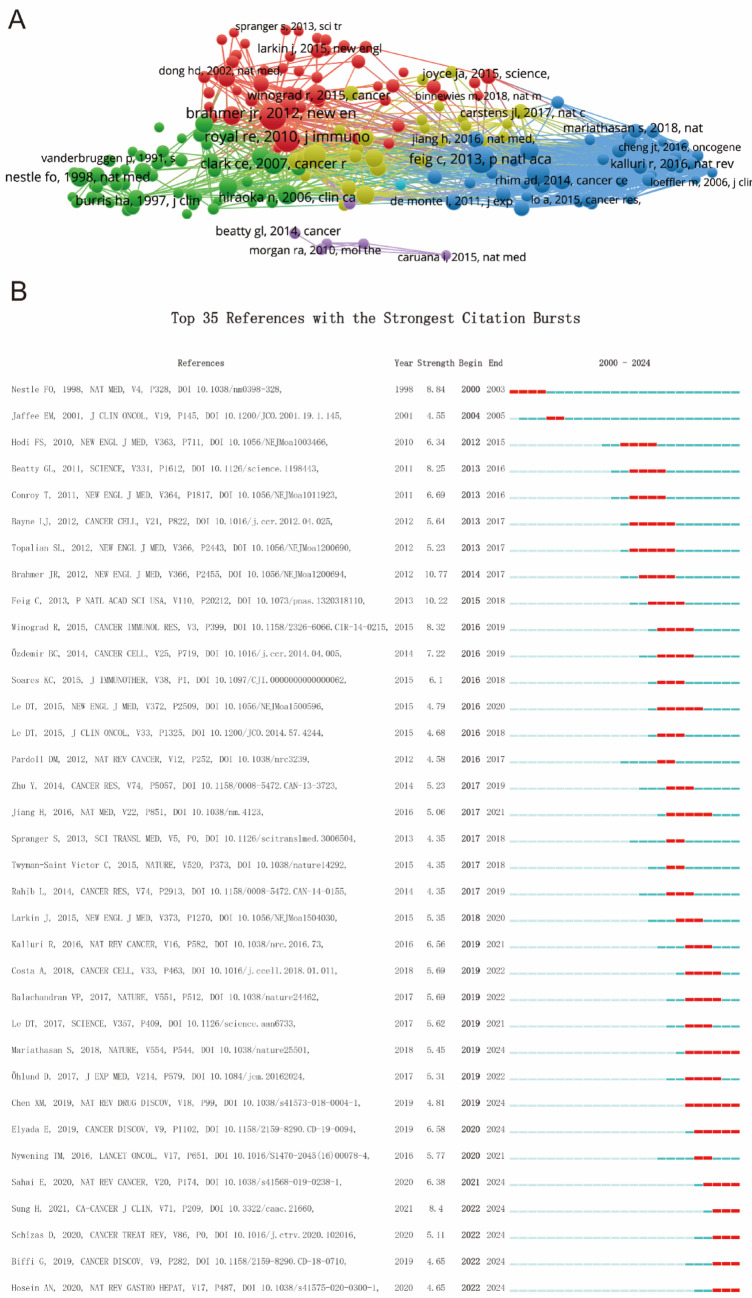
**A** Mapping of references based on Vosviewer from 2000 to 2024. **B** Top 35 references with the strongest citation bursts

Figure 7A, B illustrated the citation bursts (sudden increases in citation frequency indicating emerging hotspots or influential works) from seminal studies, those influential findings have significantly shaped the field’s development. Citation spikes, particularly in 2015–2020, highlighted periods of major breakthroughs that have driven further research. Table 6 emphasized the critical role of funding agencies in supporting this research, with the United States Department of Health and Human Services (HHS, 90 articles), National Institutes of Health (NIH, 88), and National Cancer Institute (NCI, 51) together contributing 229 publications, accounting for 43.4% of the total output, underscoring the dominant role of U.S. funding bodies. The National Natural Science Foundation of China (NSFC, 66, 12.5%) ranked third overall, highlighting Chinese strong national investment in this field. Japan also made substantial contributions, with the Ministry of Education, Culture, Sports, Science and Technology (MEXT, 29), the Japan Society for the Promotion of Science (28), and the Grants-in-Aid for Scientific Research program (25) collectively supporting 82 publications (15.6%). European support was also notable, particularly from Germany (DFG, 15; Deutsche Krebshilfe, 8) and Italy (AIRC, 12). These findings clarify that while the U.S. leads with nearly half of the global funding-supported research, strong contributions from China, Japan, and European agencies underscore the truly international nature of progress in T cell-based immunotherapy for PC.

**Table 6:** Top 10 funds related to T cell-based immunotherapy in PC from 2000 to 2024

Rank	Agencies	Article counts	Percentage
1	United states department of health and human services (HHS)	90	17.078
2	National institutes of health (NIH) USA	88	16.698
3	National natural science foundation of china (NSFC)	66	12.524
4	National cancer institute (NCI)	51	9.677
5	Ministry of education,culture,sports,science and technology	29	5.503
6	Japan society for the promotion of science	28	5.313
7	Grants-in-aid for scientific research	25	4.744
8	German research foundation (DFG)	15	2.846
9	Fondazione AIRC per la ricerca sul cancro	12	2.277
10	Deutsche krebshilfe	8	1.518

## Keyword visualization and burst test

**Fig. 8:**
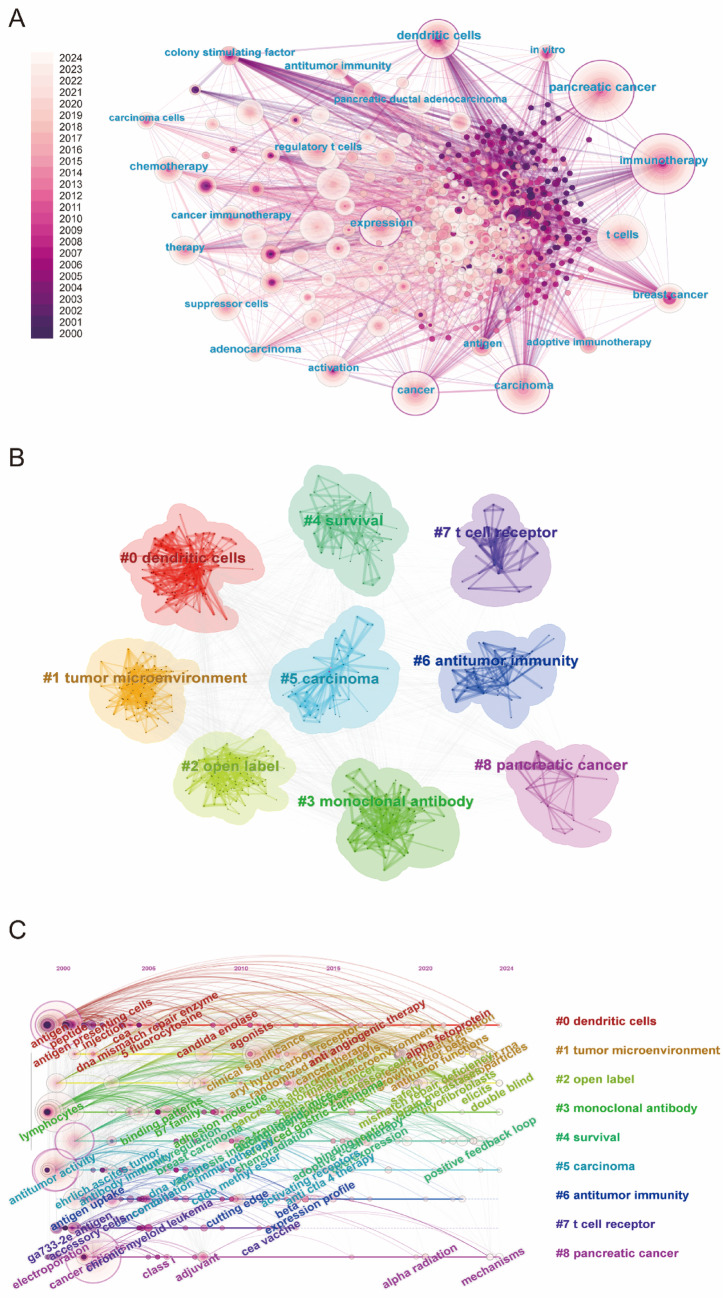
**A** Keyword network visualization produced using CiteSpace. Each node represents a keyword, with node size proportional to its frequency of occurrence. **B** Cluster information for keywords. Keywords are grouped into clusters based on co-occurrence relationships. Each cluster is displayed in a distinct color and labeled with its representative topic. **C** Keyword timeline visualization map for T cell-based immunotherapy in PC. The horizontal axis represents the chronological distribution (2000–2024). Keywords are mapped according to their first appearance and subsequent activity over time

**Fig. 9:**
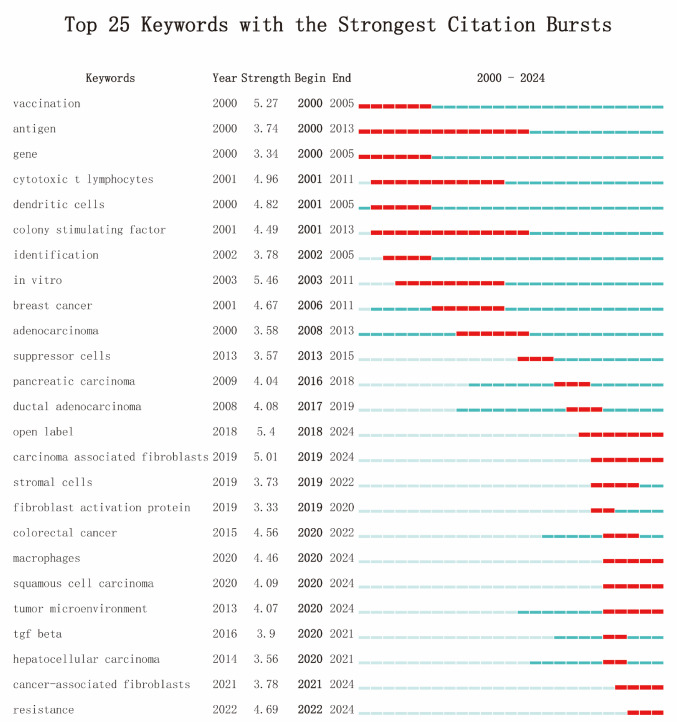
Top 25 keywords with the strongest citation bursts, arranged in ascending order based on burst time

The keyword network analysis (Fig. 8A) revealed a robust and intricate landscape of research in T cell-based immunotherapy for PC, with key terms such as PC, immunotherapy, t cells, dendritic cells, and cancer forming a dense, interconnected network. This network highlighted the central role of immune cells and therapeutic strategies targeting PC, with notable connections to adoptive immunotherapy and antitumor immunity. Cluster analysis (Fig. 8B) further refined our understanding by grouping related keywords into eight prominent research themes. These clusters included dendritic cells, tumor microenvironment, and PC, reflecting the multifaceted nature of current investigations. Additional clusters centered on monoclonal antibody, survival, and T cell receptor highlighted the increasing emphasis on targeted therapies and personalized treatment regimens. The timeline visualization (Fig. 8C) captured the temporal dynamics of these themes, illustrating the progressive shift towards studying the tumor microenvironment and its influence on the efficacy of immunotherapies. To further identify emerging trends, a burst analysis (Fig. 9) was conducted to pinpoint the keywords exhibiting the strongest increases in citation frequency. Notable bursts in pancreatic carcinoma, carcinoma-associated fibroblasts, and macrophages indicated heightened attention and growing significance in these areas. These trends suggested an evolving research focus, particularly on the cellular interactions within the pancreatic tumor microenvironment and their implications for immunotherapy. Additionally, Table 7 listed the top 10 most frequent keywords, with PC (169 occurrences), immunotherapy (141), and carcinoma (116) leading the way.

**Table 7:** The top 10 keywords with the highest frequency in T cell-based immunotherapy in PC

Rank	Keyword	Frequency	Year
1	pancreatic cancer	169	2002
2	immunotherapy	141	2000
3	carcinoma	116	2000
4	t cells	112	2000
5	cancer	97	2000
6	expression	85	2001
7	dendritic cells	74	2000
8	regulatory t cells	55	2011
9	tumor microenvironment	52	2013
10	therapy	47	2000

## Discussion

### Analysis of the geographical distribution of publications

The global landscape of T cell-based immunotherapy for cancer treatment demonstrates a significant and growing international commitment, with an increasing volume of research outputs and widespread collaboration across borders. As shown in Fig. 1A, the annual and cumulative publication outputs have consistently risen from 2000 to 2024, highlighting not only an expanding scientific interest in the field but also the substantial advancements in the development of T cell therapies. This surge in publications signifies the growing recognition of T cell-based immunotherapy in PC as a transformative approach to cancer treatment, with contributions from diverse research centers around the world. The USA has long been at the forefront of T cell-based immunotherapy in PC research, as evidenced in Table 1, where the USA accounts for approximately 35% of the total publications. This leadership is further reflected in Fig. 2B, where the USA ranks highest in terms of the H-index—a metric that integrates both publication volume and citation impact. While the USA maintains its dominant role in the field, other nations, notably China and Germany, have emerged as significant contributors, ranking second and third in publication volume, respectively. The geographical distribution of citations, depicted in Fig. 2D, reveals a nuanced landscape: although the USA leads in total citations, countries such as France, the Netherlands, and Australia outperform in terms of average citations per publication. This suggests a shift toward more specialized, high-impact research in these regions, with Australia, in particular, standing out for its applied research focus in clinically translatable studies. The varying citation patterns underscore the different research priorities across regions, with North America contributing foundational scientific studies and Europe and Oceania focusing on clinical applications and translational innovations.

The extensive international collaboration network plays a pivotal role in accelerating progress in this field. Figure 2E, F highlights the strong collaborative ties between research hubs in the USA, China, and Europe, demonstrating the interconnectedness of the global research community. These collaborations facilitate the exchange of knowledge, resources, and methodologies, fostering a collective effort to overcome the challenges of developing effective T cell therapies. This interconnected network is essential not only for advancing scientific knowledge but also for ensuring the integration of diverse perspectives that enrich the research process and enhance the global impact of discoveries. In Fig. 3C, at the institutional level, leading research centers, such as Helmholtz Association, the University of California System, and UTMD Anderson Cancer Center, continue to dominate both in terms of publication output and collaborative engagement. As illustrated in Fig. 4A, B, these institutions not only produce a significant proportion of global research but also serve as key hubs for international collaboration. Their collaborations with institutions across Europe, Asia, and other regions amplify the global reach and impact of their work, further accelerating advancements in T cell immunotherapy.

In conclusion, the bibliometric analysis of T cell-based immunotherapy in PC research underscores the dynamic and highly collaborative nature of the field. While the USA, China, and Germany are the primary contributors in terms of publication volume, countries such as France, Italy, and Australia stand out for their higher citation impact, reflecting the diversity of research approaches and focuses across regions. The expanding network of international collaborations remains a cornerstone of progress in this field, ensuring that research efforts are enhanced by a variety of perspectives and expertise. As the field of cancer immunotherapy continues to evolve, it will be shaped by the collective contributions of researchers worldwide, driven by a shared commitment to improving patient outcomes through innovative therapies and cutting-edge research.

### Analysis of journals, and studies

The global landscape of T cell-based immunotherapy in PC for cancer treatment reflects a rapidly evolving and highly specialized research field, with a clear concentration of publications in influential journals. As shown in Fig. 5A, journals such as Cancer Immunology Immunotherapy, Cancers, and Clinical Cancer Research dominate, acting as primary platforms for disseminating cutting-edge research. Other prominent journals, including Frontiers in Immunology and Journal for Immunotherapy of Cancer, reflect the growing interest in immunotherapy as a central focus of cancer treatment. The time-series data in Fig. 5B highlights the consistent rise in journal output, with publications in key journals such as Cancer Immunology Immunotherapy showing marked growth. This trend underscores the increasing prominence of T cell immunotherapy as an area of both academic and clinical interest. Bradford’s Law analysis, presented in Fig. 5C and Table 3, further illustrates the structure of knowledge dissemination, with a small number of core journals responsible for the majority of publications. This concentration suggests the establishment of a specialized and influential research community that drives the future of T cell immunotherapy. The subject categorization of T cell immunotherapy research further emphasizes its strong foundation in oncology. As shown in Table 5, Oncology stands as the leading subject category, with Immunology following closely behind, reflecting the critical intersection between cancer research and immune system therapies. Complementary fields such as Medicine, Research & Experimental, Biochemistry & Molecular Biology, and Gastroenterology & Hepatology indicate the interdisciplinary nature of the research, bridging basic science and clinical application. This diversity highlights the multifaceted approach to T cell-based immunotherapy in PC, spanning from fundamental immunological studies to clinical translational applications aimed at improving cancer treatment outcomes.

Citation network analysis, represented in Fig. 6A, provides a comprehensive view of the intellectual structure of the field, with dense interconnections among influential studies that have shaped the direction of T cell immunotherapy. The citation bursts shown in Fig. 6B identify key papers that have sparked significant attention during specific periods, marking major breakthroughs in the field. These citation surges reflect the transformative nature of research advancements, which have led to paradigm shifts in the development of new therapies and approaches to cancer treatment. Funding has played a pivotal role in advancing T cell immunotherapy, with major funding agencies such as the United States Department of Health and Human Services (HHS) and the National Institutes of Health (NIH) providing substantial support, as shown in Table 6. International agencies, including the National Natural Science Foundation of China (NSFC) and the European Research Council (ERC), also contribute significantly, reflecting the global commitment to advancing T cell-based immunotherapies. The support from these funding bodies is crucial for driving continued progress in the field and ensuring the translation of research findings into clinical applications.

In conclusion, the bibliometric analysis of T cell-based immunotherapy in PC for cancer treatment reveals a well-established, interdisciplinary research domain. The concentration of research in high-impact journals, the ongoing rise in publication output, and the support from leading funding agencies point to a rapidly advancing field poised for significant clinical impact. As research continues to evolve, these insights provide valuable guidance for future directions and emerging opportunities in the development of T cell therapies for cancer treatment.

### Analysis of research hotspots

The landscape of T cell-based immunotherapy for PC treatment has evolved significantly, as highlighted by dynamic keyword clustering, citation bursts, and temporal shifts in research focus. The keyword co-occurrence network (Fig. 8A) reveals strong interconnections between core research areas such as “T cells,” “cancer immunotherapy,” and the “tumor microenvironment,” suggesting an integrated approach to cancer treatment. Cluster decomposition analysis (Fig. 8B) identifies distinct and emerging research domains, while the timeline visualization (Fig. 8C) tracks the evolving nature of these areas over time. Citation burst analysis (Fig. 9) further highlights high-impact topics, including “pancreatic cancer” and “immunotherapy,” marking them as central to future research trajectories.

#### T cell immunotherapy: advancements and optimization

T cell-based immunotherapy in PC has solidified its position as a cornerstone of cancer treatment, reflected in its dominance in keyword rankings and citation bursts (Figs. 8 and 9). Immunotherapy leverages the immune system, particularly T cells, to target and eliminate cancer cells with precision (Oliveira and Wu 2023). This approach has gained widespread attention due to its potential to significantly improve cancer treatment outcomes, particularly for hard-to-treat cancers.

Recent advancements focus on optimizing T cell activation, expansion, and targeting (Perica et al. 2014; Wu et al. 2020a). T cells, when activated appropriately, have the capacity to recognize and attack tumor cells presenting specific antigens. However, their therapeutic potential is often hindered by factors such as the immunosuppressive TME and tumor heterogeneity (Falcomatà et al. 2023; Dagogo-Jack and Shaw 2018). Therefore, current research aims to enhance the effectiveness of T cells by engineering them to better recognize tumor markers and by expanding them ex vivo before reinfusion. The increasing prominence of terms such as “T cell receptor” and “adoptive immunotherapy” underscores the growing focus on these strategies. Adoptive T cell therapy, particularly Chimeric Antigen Receptor T cell (CAR-T) therapy, has already achieved remarkable success in treating hematologic cancers like leukemia and lymphoma (Zhang et al. 2022; Hamilton et al. 2024; Leick et al. 2022). These approaches are increasingly being explored in solid tumors, where barriers such as limited intratumoral trafficking and TME-mediated immunosuppression remain major hurdles. Continued advances in CAR-T engineering and refinements to adoptive T-cell strategies point to a future in which T-cell immunotherapy could become a broadly effective option across multiple cancer types.

PC-specific clinical efforts have tested engineered T-cell therapies. In a Phase I study of transient mRNA mesothelin-directed CAR-T cells, 2 of 6 patients with chemotherapy-refractory PC achieved stable disease with marked metabolic responses and acceptable safety (Beatty et al. 2018). A pilot trial evaluating co-infusion of mesothelin- and CD19-targeted CAR-T cells reported feasibility and disease stabilization in 1 of 3 metastatic PC patients (Ko et al. 2020). For TCR therapy, a NEJM case report documented objective regression of metastatic PC following infusion of KRAS^G12D-specific TCR-engineered T cells (Simnica and Kobold 2022). For TIL therapy, the field has reached a regulatory milestone: in 2024, the US FDA approved lifileucel (Amtagvi) for unresectable or metastatic melanoma following prior PD-1 therapy. While not yet standard in PC, TIL-based therapy is under prospective evaluation in PC cohorts within Phase I/II trials (e.g., NCT03935893; NCT05098197), although definitive efficacy signals remain pending. Together, these PC-relevant clinical data substantiate the approach’s clinical relevance while underscoring the need for larger, dedicated trials to define patient selection, dosing, and risk–benefit profiles.

#### Tumor microenvironment (TME) and immunosuppressive mechanisms

The TME plays a critical role in the success or failure of T cell-based therapies, as reflected by its continued prominence in both network analysis (Fig. 8A, B) and citation bursts (Fig. 9). The TME is composed of a variety of components-including immune cells, stromal cells, and extracellular matrix elements-that not only support tumor growth but also create an immunosuppressive environment (Wu et al. 2022, 2020b). This immune suppression can inhibit the ability of T cells to effectively target and destroy cancer cells.

To improve T cell therapy outcomes, significant research is focused on overcoming the immunosuppressive TME. Strategies aimed at modifying the TME include targeting immune checkpoint inhibitors like PD-1 and CTLA-4 (Kumagai et al. 2022; Marangoni et al. 2021). These immune checkpoints act as “brakes” on the immune system, and blocking them has led to major breakthroughs in cancer treatment, particularly for melanoma and non-small cell lung cancer (Kleffel et al. 2015; Cheng et al. 2024). The combination of immune checkpoint inhibitors with T cell therapies is showing great promise, enabling T cells to overcome TME-induced suppression. The growing presence of keywords such as “immune checkpoints” and “tumor microenvironment” signifies an increasing recognition of the need to address the TME in the development of effective cancer immunotherapies. Additionally, other novel approaches, including the use of engineered nanoparticles and oncolytic viruses to modify the TME, are being explored to enhance immune responses and improve treatment efficacy (Liu et al. 2023). These innovations underscore the importance of addressing the TME as a central challenge in T cell-based cancer therapies.

#### Precision immunotherapy: tumor antigens and personalized approaches

The identification and targeting of tumor antigens are another burgeoning area in T cell-based immunotherapy in PC, as evidenced by the growing prominence of terms such as “tumor antigens,” “monoclonal antibodies,” and “personalized therapy” (Figs. 8 and 9). Tumor-specific antigens are proteins or molecules expressed predominantly on cancer cells, making them ideal targets for immune-mediated destruction (Fan et al. 2023). Precision immunotherapy seeks to harness these tumor-specific markers to design more targeted and effective treatments, thereby minimizing off-target effects and improving therapeutic outcomes.

Monoclonal antibodies play a key role in precision immunotherapy, enhancing T cell targeting by either blocking immune suppressive signals (such as immune checkpoint inhibitors) or directly targeting tumor antigens (Zhao et al. 2021). The growing interest in “monoclonal antibodies” and “personalized therapy” in the keyword rankings underscores the importance of this strategy. For example, engineered T cells, such as CAR-T cells, can be designed to recognize specific tumor antigens, offering a highly targeted treatment option (Hong et al. 2020; Tieu et al. 2024). Additionally, the combination of monoclonal antibodies with T cell therapies is a promising avenue for improving both the specificity and efficacy of cancer treatments. Research is increasingly focused on identifying novel tumor antigens that could serve as targets for immunotherapy. As these antigens are identified and validated, the potential for personalized T cell-based therapies to treat a broad range of cancers will continue to expand.

### Future research trends

The future of T cell-based immunotherapy for PC treatment is set to undergo a transformation, with rapid advancements in immunology, bioengineering, and precision medicine. The insights drawn from keyword networks (Fig. 8A), cluster analyses (Fig. 8B), and citation burst data (Fig. 9) suggest that the next phase of cancer immunotherapy will focus on a convergence of innovative technologies and approaches. These include the enhancement of T cell therapies through novel modifications, the integration of immune-modulating agents, and the development of patient-specific, personalized therapies. These trends highlight a paradigm shift toward more adaptive, biologically integrated, and precisely targeted therapeutic strategies aimed at improving cancer treatment outcomes.

#### T cell modulation and enhancement technologies

A key focus in the future of T cell immunotherapy is the development of novel T cell modulation technologies designed to optimize T cell functionality, enhance their persistence, and overcome the challenges posed by immune evasion mechanisms in tumors. The increasing prominence of terms such as “T cell receptor”, “adoptive immunotherapy”, and “tumor antigens” in the keyword network (Fig. 8A, B, and Table 7) signals the continued focus on improving the specificity, activity, and durability of T cell therapies.

Advances in gene editing technologies, such as CRISPR-Cas9, are enabling the creation of highly specialized T cells that can bypass the immunosuppressive effects of the TME (Schmidt et al. 2022; Chen et al. 2021). By genetically modifying T cells, scientists can enhance their tumor-targeting abilities, increase their resistance to immune checkpoint inhibitors, and improve their persistence in the body, thus maximizing their therapeutic potential. This is particularly critical for treating solid tumors, which are known for being more resistant to conventional T cell therapies.

Further innovations in TCR engineering will also play a crucial role (Baulu et al. 2023; Zhao et al. 2022; Isshiki et al. 2025). Researchers are working to enhance the ability of T cells to recognize tumor-specific antigens with higher precision. In parallel, immune modulation strategies aimed at overcoming the suppressive effects of regulatory T cells (Tregs) and other components of the TME are gaining traction (Zhang et al. 2024; Goverman 2021). The ability to “reprogram” the TME to make it more conducive to T cell activation is essential for improving the outcomes of T cell therapies. The use of immune checkpoint inhibitors like anti-PD-1 and anti-CTLA-4 has already proven successful in boosting T cell responses, and combining these therapies with engineered T cells may become a standard strategy to enhance cancer immunotherapy (Kumagai et al. 2022). As “antitumor immunity” continues to gain recognition in citation bursts (Fig. 9), combining immune checkpoint blockade with T cell modulation will likely be a central approach in future therapeutic regimens.

#### Integration of immune modulation agents and cancer vaccines

Another key trend in the evolution of T cell immunotherapy is the integration of immune modulation agents and cancer vaccines with T cell-based treatments. As shown in the network and citation burst data (Fig. 8A, B, and Table 7), the combination of T cell therapies with other immune-enhancing agents such as monoclonal antibodies, tumor vaccines, and adjuvants will drive the next wave of innovation in cancer immunotherapy. The integration of these therapies aims to stimulate a robust and targeted immune response against cancer cells, while minimizing systemic side effects and improving the overall efficacy of treatment.

The development of cancer vaccines that specifically target tumor antigens and stimulate T cells is expected to become a prominent area of research (Waldman et al. 2020; Sethna et al. 2025). These vaccines can help train the immune system to recognize cancer-specific markers, triggering a powerful immune response that targets and eliminates cancer cells. Combining vaccines with immune checkpoint inhibitors can further enhance the activity of T cells, providing a synergistic effect that is likely to improve treatment outcomes. Moreover, adjuvants-substances that enhance the immune response to a vaccine-will play an important role in improving the potency of cancer vaccines.

#### Personalized and precision immunotherapy

Personalized medicine is the future of T cell-based immunotherapy in PC, as it aims to design treatment strategies tailored to the unique genetic and molecular profile of each patient’s tumor. The sustained prominence of terms such as “personalized therapy”, “tumor microenvironment”, and “targeted therapies” in keyword rankings (Fig. 8A, B, and Table 7) signals a shift toward highly individualized therapeutic regimens. Research in this area will focus on profiling individual tumors to identify specific mutations, biomarkers, and antigens that can be targeted by T cell therapies (Cao et al. 2022).

Next-generation sequencing (NGS) and liquid biopsy technologies will be at the forefront of this transformation, enabling the rapid identification of tumor-specific mutations and the design of customized therapies that target those alterations (Mosele et al. 2024; Loy et al. 2024). By leveraging artificial intelligence (AI) and machine learning, oncologists will be able to predict which T cell therapy regimens are most likely to be effective based on the individual patient’s tumor characteristics (Perez-Lopez et al. 2024; Keyl et al. 2025). These AI-driven platforms will analyze vast amounts of clinical and genetic data to recommend the optimal treatment strategies, enhancing both efficacy and safety.

#### Epigenetic modulation to enhance T-cell-based immunotherapy

Epigenetic regulators shape tumor antigenicity and T-cell fate. Dual or selective EZH1/EZH2 inhibition can reprogram tumors toward an immunogenic phenotype and potentiate CAR-/TCR-engineered T cells; in parallel, transient EZH2 inhibition during ex vivo expansion preserves T-cell stemness and improves ACT efficacy, including in combination with PD-1 blockade (Porazzi et al. 2025; Hou et al. 2025). These data nominate epigenetic–immunotherapy combinations as testable strategies in PC to overcome poor antigenicity, stromal exclusion, and myeloid-dominant suppression.

#### Broader clinical context: supportive care

Although liver and peritoneal dissemination predominate in PC, bone metastases-while relatively uncommon-can be clinically devastating and under-recognized. Case-based syntheses indicate that bone-targeted agents such as zoledronic acid may warrant exploration for skeletal protection and symptom control in this population, extrapolating from broader oncology experience (Argentiero et al. 2019). These agents are not standard of care in PC, and prospective PC-specific data remain limited; nevertheless, integrating bone health assessment and considering anti-resorptive strategies in selected patients may open supportive avenues within comprehensive care pathways. Future work should clarify patient selection, dosing schedules, and risk–benefit profiles in PC cohorts (Sanford et al. 2013).

The future of T-cell-based immunotherapy for cancer treatment is set to undergo significant advancements, with several key areas driving this transformation. Research will focus on enhancing T-cell therapies through novel modulation technologies designed to optimize T-cell functionality, increase their persistence, and overcome immune evasion mechanisms employed by tumors. The integration of immune-modulating agents, such as immune checkpoint inhibitors and cancer vaccines, will further enhance the overall efficacy of T-cell therapies by promoting more robust and targeted immune responses (Peng et al. 2025). At the same time, personalized and precision immunotherapy will allow treatments to be tailored to individual tumor profiles, utilizing advanced technologies like next-generation sequencing, liquid biopsy, and artificial intelligence to optimize therapy selection (Basiri 2023). Additionally, epigenetic modulation will emerge as a promising approach to enhance T-cell activity, potentially improving outcomes in challenging cancers. Moreover, broader clinical contexts will focus on improving patient quality of life by exploring supportive care strategies that complement cancer treatments (Villanueva et al. 2020). Collectively, these research directions highlight a paradigm shift toward more adaptive, biologically integrated, and precisely targeted therapeutic strategies that will not only improve cancer treatment outcomes but also move beyond conventional, one-size-fits-all approaches, offering renewed hope for cancer patients.

### Limitations of this study

This bibliometric analysis offers valuable insights into the evolving landscape of T cell-based immunotherapy for PC. However, several limitations must be acknowledged. A fundamental challenge remains the incomplete understanding of how engineered T cells interact with the highly immunosuppressive pancreatic tumor microenvironment. The precise molecular mechanisms regulating T cell infiltration, persistence, and functional exhaustion within these tumors are not yet fully elucidated, posing a barrier to the rational design of more effective and durable immunotherapies. Bridging these gaps is essential for optimizing next-generation strategies that enhance antitumor immunity while overcoming resistance mechanisms.

Methodologically, this study is subject to potential selection biases due to database constraints and language restrictions. The exclusion of key repositories such as PubMed, Cochrane, and Embase, as well as non-English literature, may have limited the comprehensiveness of the analysis. Although Sect. 3.5 briefly acknowledges these issues, the study does not include a detailed comparison of publication counts across databases or a more in-depth exploration of their impact, which could have further clarified the representativeness of the findings. Additionally, reliance on citation frequency as a primary metric may undervalue recently published, high-impact studies that have not yet accumulated extensive citations, potentially skewed bibliometric trends and underrepresenting emerging innovations in T cell therapies for PC. Potential tool-specific biases introduced by VOSviewer and CiteSpace were not explicitly discussed. The algorithms and weighting schemes used by these visualization tools may influence clustering patterns and network representations, but the current analysis only addresses general methodological limitations without detailing such tool-related constraints. Furthermore, the visualization and clustering results may be influenced by the inherent algorithms and weighting schemes applied in tools such as VOSviewer and CiteSpace, which could introduce additional bias.

To provide a more comprehensive and representative analysis, future studies should integrate a broader range of databases and multilingual sources. Moreover, refining bibliometric methodologies to account for emerging but under-cited research could yield a more accurate depiction of advancements in the field. Future efforts should also explicitly address potential biases introduced by visualization tools such as VOSviewer and CiteSpace, ensuring that algorithmic and weighting-related limitations are transparently acknowledged. Such efforts will be instrumental in accelerating the development of engineered T cell therapies with enhanced tumor infiltration, resistance to immunosuppressive signaling, and improved clinical efficacy, ultimately contributing to more effective treatment strategies for PC.

## Conclusion

This bibliometric analysis reveals significant global trends and a growing research emphasis on T cell-based immunotherapy for pancreatic cancer from 2000 to 2024. The USA has emerged as the dominant contributor, exhibiting high citation frequencies and H-index scores, underscoring its pivotal role in advancing this field. This leadership reflects sustained investment in immunotherapy research, cutting-edge clinical developments, and interdisciplinary collaborations aimed at improving treatment outcomes for this highly aggressive malignancy. Our findings also highlight key directions that are expected to shape the future of T cell-based therapies for pancreatic cancer. These include the development of engineered T cells with enhanced persistence, tumor infiltration, and resistance to immunosuppressive signals within the pancreatic tumor microenvironment. Additionally, integrating gene-editing technologies and synthetic biology approaches offers new avenues for optimizing therapeutic efficacy. Advances in personalized and precision immunotherapies-leveraging biomarker-driven patient selection, combinatorial treatment strategies, and novel delivery platforms-are poised to further enhance clinical responses and broaden the applicability of T cell therapies. Collectively, these emerging research trajectories will be instrumental in overcoming existing challenges, refining immunotherapeutic strategies, and unlocking novel opportunities to improve efficacy, durability, and patient outcomes in pancreatic cancer treatment.

## Experimental section

### Data source

For this bibliometric analysis, the Web of Science Core Collection (WOSCC) was chosen as the primary data source due to its comprehensive coverage and methodological rigor. T cell-based immunotherapy for pancreatic cancer is a highly interdisciplinary field, spanning oncology, immunology, molecular biology, bioengineering, and clinical medicine. A robust bibliometric study requires an integrated database that captures research across these domains to provide an accurate and representative assessment of global trends. WOSCC offers several key advantages. First, its inclusion of cited reference data enables in-depth knowledge mapping, facilitating a clearer understanding of research interconnections and the evolution of immunotherapeutic strategies. Second, its citation reports serve as validation tools, ensuring the accuracy, reliability, and reproducibility of bibliometric analyses. Third, WOSCC datasets are natively compatible with leading bibliometric software, eliminating the need for format conversion and minimizing risks of data loss or corruption, thereby preserving analytical integrity. Furthermore, WOSCC encompasses the Science Citation Index Expanded (SCIE), which maintains stringent quality control by indexing rigorously vetted, high-impact journals. Additionally, its journal selection process aligns with Bradford’s Law and Garfield’s Law, ensuring that core publications are effectively captured while minimizing omissions.

### Retrieval strategy

T cell-based immunotherapy for pancreatic cancer is a rapidly evolving field, integrating advances in cellular engineering, tumor immunology, and precision medicine. Conducting a bibliometric analysis in such a dynamic research landscape requires a carefully designed retrieval strategy to capture both established and emerging trends while minimizing selection biases. A narrowly defined search strategy focusing solely on widely recognized terms related to T cell immunotherapy risks overlooking novel yet impactful contributions, particularly those at the intersection of synthetic biology, immune checkpoint modulation, and personalized therapy. Conversely, an overly broad approach may inflate the prominence of well-established research clusters, reinforcing existing publication biases and potentially skewing the representation of emerging innovations. To address these challenges, we employed a retrieval framework that balances specificity and inclusivity, ensuring a comprehensive yet nuanced assessment of global research trends. Our objective was to map the evolving research landscape, identifying key developments that make this analysis relevant to scientists, clinicians, and industry leaders advancing immunotherapeutic strategies for pancreatic cancer.

**Fig. 10:**
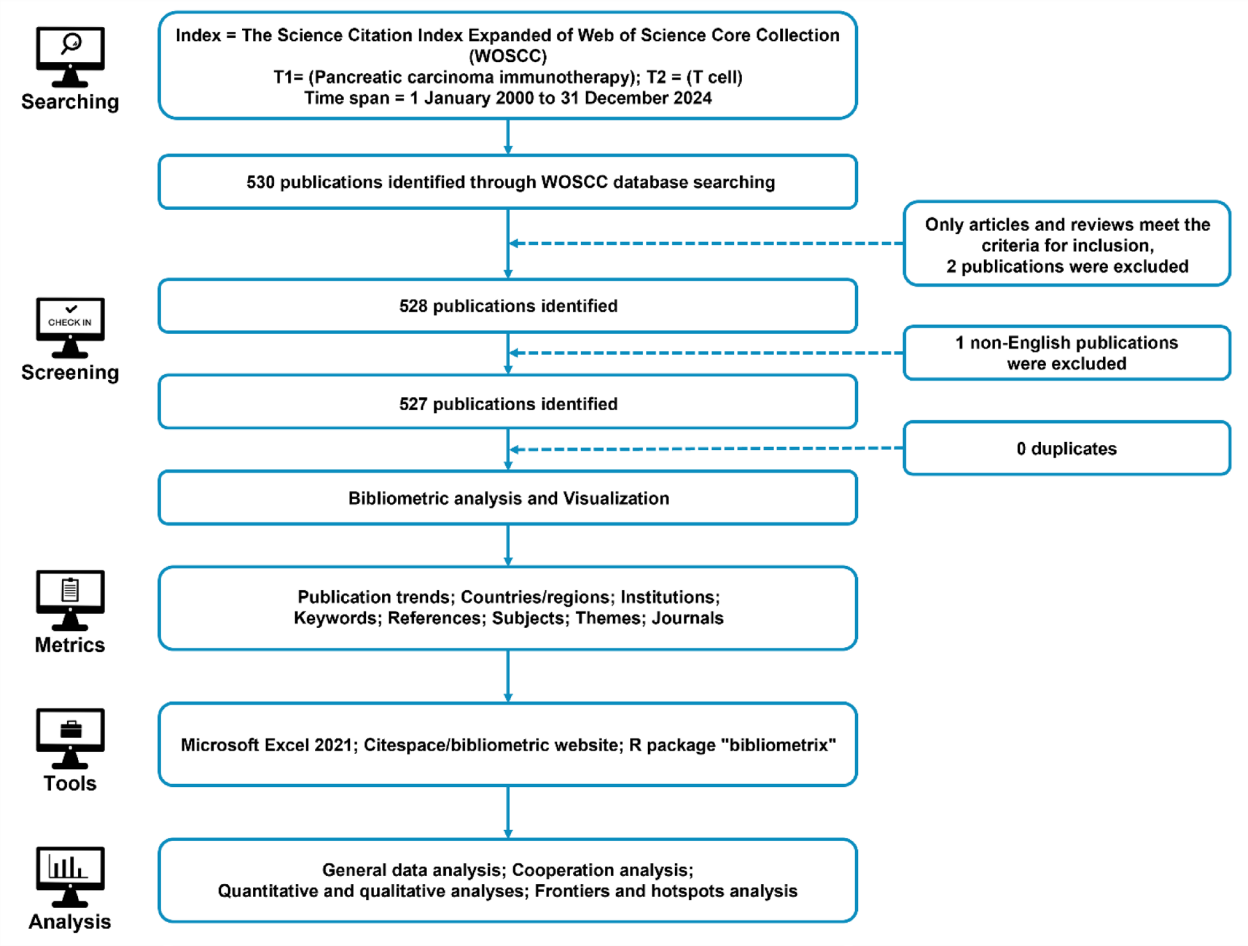
A detailed flowchart of the search and selection process, ensuring a systematic and comprehensive bibliometric evaluation. The initial search, applying restrictions on search terms and publication dates, yielded 530 publications. Further refinement based on publication type (Article and Review) and language (English) resulted in a final dataset of 527 publications selected for bibliometric analysis. This dataset spans seven key analytical dimensions: publication trends, countries/regions, institutions, keywords, references, research disciplines, and journals

The retrieval criteria were structured as follows: Index = Science Citation Index Expanded (SCIE) of the Web of Science Core Collection (WOSCC); Search Terms = (“Pancreatic carcinoma immunotherapy” [T1]) AND (“T cell” [T2]). The publication time span encompassed research published between January 1, 2000, and December 31, 2024. To ensure methodological consistency, only Article and Review document types were included, with the language restricted to English to maintain uniformity in bibliometric analysis. A detailed overview of the retrieval and screening process is provided in Fig. 10 illustrating the methodological framework used to construct a representative and high-quality dataset.

### Bibliometric analysis and visualization

To ensure a rigorous and comprehensive bibliometric analysis of T cell-based immunotherapy for pancreatic cancer, publication and citation data were directly retrieved from the Web of Science Core Collection (WOSCC). Data processing and statistical analysis were conducted using GraphPad Prism 8.0 and Microsoft Excel 2021, while the global distribution of research were generated from WOSCC data and bibliometric software, and subsequently formatted in PowerPoint for visualization, effectively capturing publication trends and geographic research distribution over the two-decade study period. To evaluate national contributions, total publication output from leading countries was extracted and analyzed using WOSCC, R “bibliometrix” 5.0, and GraphPad Prism 8.0. The H-index was calculated at the country/region level to evaluate the collective academic impact, providing deeper insights into research influence and leadership across different geographic areas. Network-based bibliometric mapping was performed using VOSviewer 1.6.20, which facilitated the construction and analysis of co-citation, co-occurrence, and bibliographic coupling networks, offering a structured assessment of research connectivity and thematic evolution. Additionally, inter-country collaboration patterns were visualized using Microsoft PowerPoint 2021, enhancing the depiction of global research networks. To detect citation bursts, keyword trends, and reference clustering, we employed CiteSpace (version 6.4), enabling the identification of emerging research trajectories and thematic clusters. This integrative approach provided a high-resolution analysis of evolving research priorities, highlighting key advances and intellectual milestones in T cell-based immunotherapy for pancreatic cancer.

By integrating GraphPad Prism 8.0, Microsoft Excel 2021, VOSviewer, R “bibliometrix,” and CiteSpace, this study conducted a methodologically robust bibliometric assessment, offering a systematic and data-driven perspective on global research output, citation dynamics, and scholarly collaborations. These analytical tools ensured a precise and in-depth evaluation of the field’s development, shedding light on its trajectory and future directions.

## Data Availability

The data that support the findings of this study were obtained from the Web of Science Core Collection database. Access to these data requires subscription and permission from Clarivate Analytics. The raw data are not publicly available but can be accessed through the Web of Science platform subject to its licensing terms.
